# Disparities in the quality of care for chronic hepatitis C among Medicare beneficiaries

**DOI:** 10.1371/journal.pone.0263913

**Published:** 2022-03-10

**Authors:** Linh Tran, Jeah Jung, Roger Feldman, Thomas Riley

**Affiliations:** 1 Department of Health Policy and Administration, College of Health and Human Development, Pennsylvania State University, University Park, Pennsylvania, United States of America; 2 Division of Health Policy and Management, School of Public Health, University of Minnesota, Minneapolis, Minnesota, United States of America; 3 Department of Medicine, College of Medicine, Pennsylvania State University, Hershey, Pennsylvania, United States of America; Centers for Disease Control and Prevention, UNITED STATES

## Abstract

**Purpose:**

Chronic hepatitis C virus (HCV) infection is an important public health concern. Limited information exists on disparities in the quality of HCV care. We examine disparities in genotype or quantitative HCV ribonucleic acid testing before and after starting HCV treatment, and screening for hepatocellular carcinoma (HCC) in HCV patients with cirrhosis.

**Methods:**

This national study included Medicare beneficiaries with HCV between 2014 and 2017. We used bivariate probit to estimate the probability of receiving recommended tests before and after HCV treatment by patient race/ethnicity, urban/rural residence, and socioeconomic status. We used multivariate logistic regression to estimate adjusted odds ratios (aOR) of HCC screening among beneficiaries with cirrhosis by patient factors.

**Findings:**

Of 41,800 Medicare patients with HCV treatment, 93.47% and 84.99% received pre- and post-treatment testing. Patients in racial minority groups had lower probabilities of pre- and post-treatment testing than whites. Rural residents were less likely to receive a post-treatment test (Coef. = -0.06, 95% CI: -0.11, -0.01). Among HCV patients with cirrhosis, 40% (24,021) received at least one semi-annual HCC screening during the study period. The odds of HCC screening were 14% lower in rural than in urban patients (aOR = 0.86, 95% CI: 0.80, 0.92), lower in African Americans (aOR = 0.93, 95% CI: 0.90, 0.96), but higher among Hispanics than in whites (aOR = 1.09, 95% CI: 1.04, 1.15). There was no significant association between ZIP-level income or education and HCC screening.

**Conclusions:**

Disparities in the quality of HCV care existed by patient race/ethnicity, urban/rural residence, and socioeconomic status. Continued efforts are needed to improve the quality of care for all HCV patients—especially rural patients and racial/ethnic minorities.

## Introduction

Chronic hepatitis C virus (HCV) infection is an important public health concern in the US, with 2.7–3.9 million people chronically infected [1]. Left untreated, HCV can cause liver complications such as cirrhosis, hepatocellular cancer, and liver damage [2]. In addition, patients with chronic HCV have increased risk of developing extrahepatic manifestations, which lead to or aggravate other conditions, such as rheumatoid arthritis, cardiovascular diseases, and diabetes [3, 4].

Many organizations and professional societies have developed guidelines for HCV care, with recommendations covering diagnosis, management, and treatment of HCV [5–7]. Due to the burden of HCV infection and related diseases, the Centers for Medicare and Medicaid Services (CMS) has identified HCV infection as a priority area for quality measurement [8]. To promote engagement in HCV care, CMS has used several HCV quality-of-care measures in the Physician Quality Reporting System (PQRS)–a program that provides financial incentives for health care professionals to measure and report specified quality indicators to Medicare [8].

Despite these guidelines, evidence suggests that systematic deficiencies in quality of HCV care exist. For example, patients in the Veteran’s Administration (VA) healthcare network were less likely to receive recommended care if they were older, single, without advanced liver disease, or seen by primary care physicians [8, 9]. Race/ethnicity was also associated with lower use of recommended HCV care and treatment [10–12]. A study of patients in the Northwest Network of the VA found that Blacks were significantly less likely to have their virus genotyped, undergo complete laboratory evaluation, and receive antiviral treatment than whites [10].

Evidence on rural/urban and socioeconomic disparities in quality of HCV care is limited. Rural residents often encounter barriers that limit their ability to obtain recommended care, such as insufficient numbers of physicians, distance, and transportation [13]. Hence, quality of HCV care may be lower for rural than for urban patients. Moreover, socio-economically disadvantaged populations generally have a lower probability of receiving optimal care [14–21].

Recently developed direct-acting antiviral (DAA) treatments for HCV (the first DAA, sofosbuvir, was approved in the U.S. on December 1, 2013) have over 90% cure rates, much higher than 50% for the former interferon-based treatment [2]. With the availability of highly effective DAA therapy, it is important to ensure that HCV patients receive appropriate testing and treatment. While differences in DAA use across patient groups are well documented [9–11, 22], few studies have evaluated factors associated with quality of HCV care, especially services to monitor patients who have initiated DAA treatment. Understanding factors that impact how well HCV patients engage in pre-treatment and post-treatment procedures is critical to improve clinical quality and health outcomes.

Existing studies of disparities in quality of care for HCV have focused on a single measure and a limited range of factors associated with poorer quality [9–12]. Moreover, these studies mostly used data from small samples and simulation models [23–29]. We extend the prior research by examining disparities in quality of HCV care using national Medicare administrative claims data. Medicare is a federal health insurance program in the U.S. for people aged 65 years and older, for younger patients with disability and those with end-stage renal disease or amyotrphic lateral slerosis. Because a large proportion of HCV-infected individuals in the US were born between 1945 and 1964 [30], Medicare covers the group with the highest prevalence of HCV. Thus, examining disparities in quality of HCV care among Medicare beneficiaries is important. In addition to examining national data, we measured quality of HCV care using several indicators from the Medicare PQRS (S1 Table) and/or medical organization guidelines [31]. Finally, we examined a wider range of factors associated with disparities in quality of HCV care, including patients’ rural/urban, racial/ethnic, and socioeconomic status.

## Methods

### Data sources

We used Medicare claims from 2013 through 2017 for inpatient, skilled nursing facility, outpatient, and physician services to identify HCV patients and the presence of cirrhosis. The 2013 files were used only to ensure that patients did not seek HCV care during that year. Medicare Master Beneficiary Summary Files (MBSF) were used to obtain patient demographic information and indicators of health risks. DAA treatment from 2014 to 2017 was identified from Medicare Part D (coverage for outpatient prescription drugs) data. The American Community Survey (ACS) supplied information on ZIP-level education and income and state-level total and rural population aged 45 and over. The Area Health Resources Files (AHRF) provided information on county-level specialist density. Rural-Urban Commuting Area (RUCA) codes were used to assign urban-rural status to each patient based on his/her ZIP code of residence. Institutional Review Board approval from the Pennsylvania State University was obtained prior to the study. Informed consent was waived because no data were collected directly from human subjects. All patient identifiers were stripped off before the authors accessed the data. Only scrambled patient identifiers—i.e., fake numbers—were provided to the authors.

### Patient selection

We selected beneficiaries in traditional fee-for-service (FFS) Medicare who newly sought HCV care between January 1, 2014 and December 31, 2017 after a one-year washout period. This criterion identified patients who were at a relatively similar stage of seeking HCV care. HCV was defined by the standard algorithm used by the Centers for Medicare and Medicaid Services (CMS) [32]: at least 1 inpatient or skilled nursing facility claim, or at least 2 hospital outpatient or carrier (physician) claims of HCV in a given year. HCV claims were identified by International Classification of Diseases (ICD) codes: ICD-9 codes of 070.44, 070.54, 070.70, 070.71 or ICD-10 codes of B18.2, B19.20, B19.21.

Patients enrolled in Medicare Advantage (the program that provides Medicare benefits through private health insurers) were excluded because we did not have claims for them. Beneficiaries with missing information on RUCA codes, ACS and AHRF variables were also excluded.

**Fig 1:**
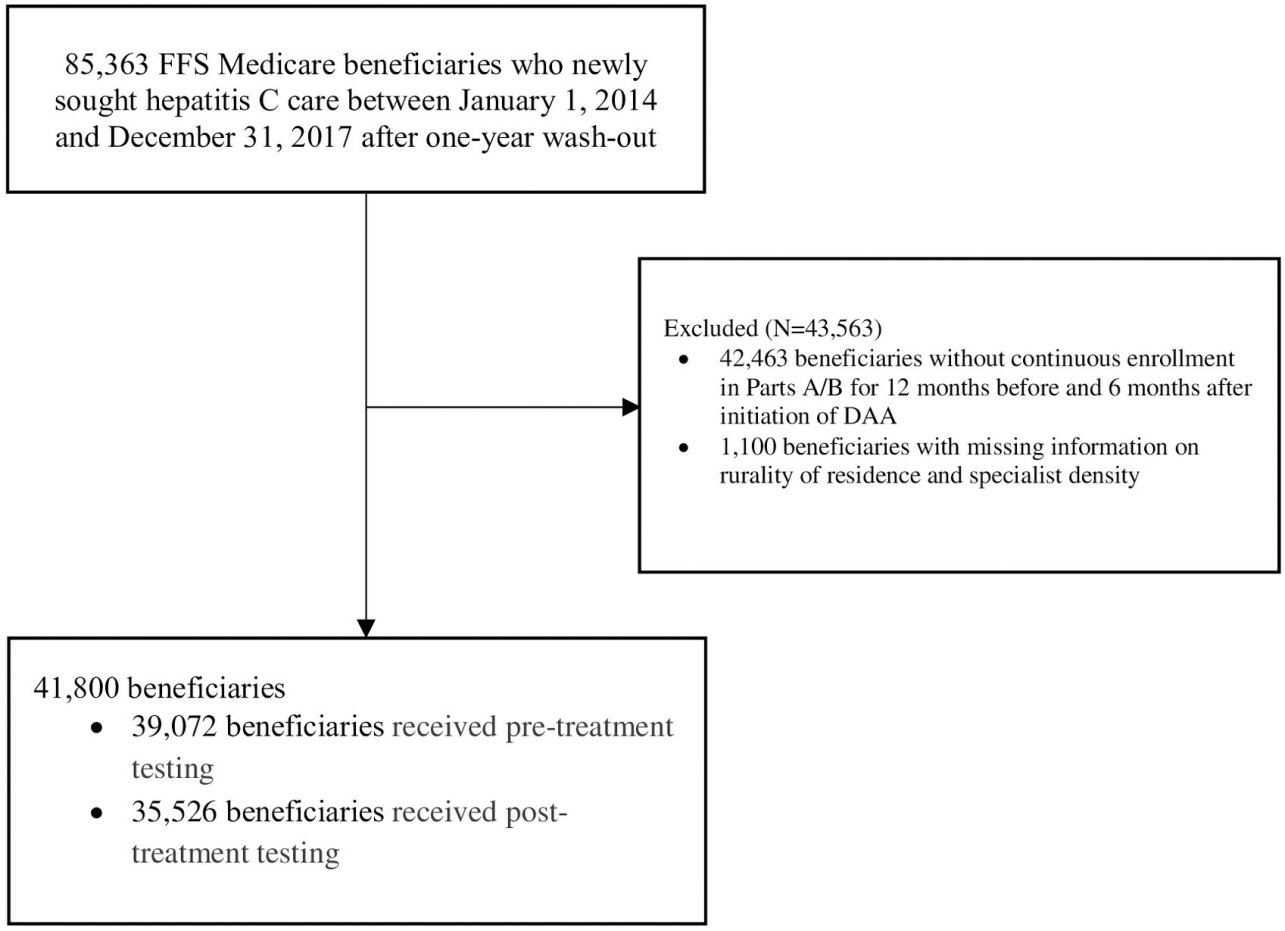
Study sample selection for pre- and post-treatment testing in DAA users. Abbreviations: FFS, fee-for-service.

We created two samples from these patients. The first sample was beneficiaries who initiated DAAs during the study period. Patients were required to be continuously enrolled in Part A (coverage for hospital and post-acute care services) and Part B (coverage for outpatient medical care) for 12 months before and 6 months after the initiation of DAA. DAA use was defined as filling at least one prescription for one of the following DAAs between 2014 and 2017: elbasvir/grazoprevir, ledipasvir/sofosbuvir, ombitasvir/paritaprevir/ritonavir plus dasabuvir, sofosbuvir, sofosbuvir/velpatasvir, sofosbuvir/velpatasvir/voxilaprevir, or glecaprevir/pibrentasvir. No patient was included more than once in our study sample as only the most recent treatment initiation was counted. Fig 1 presents a diagram of the first sample selection process.

**Fig 2:**
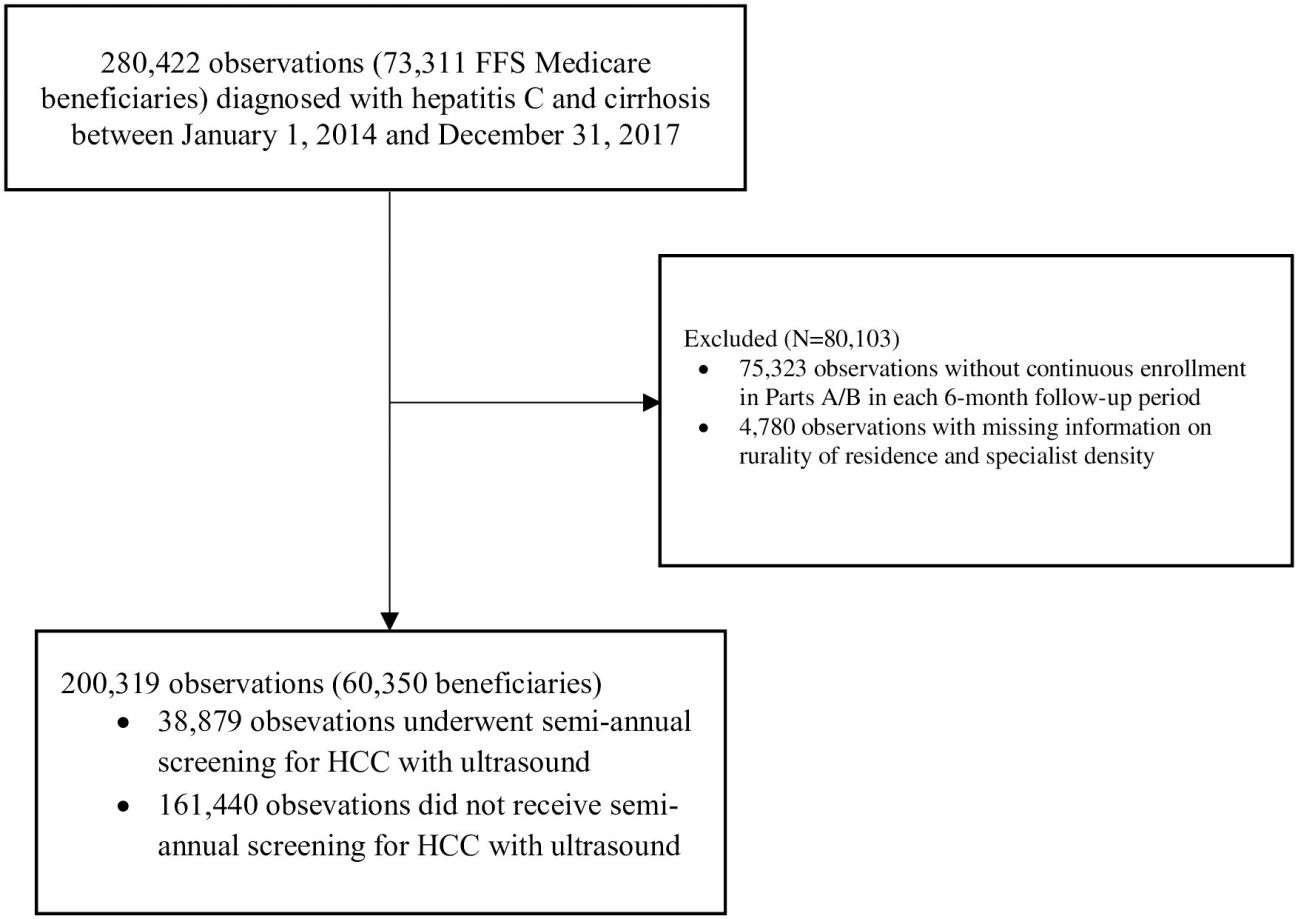
Study sample selection for routine screening for hepatocellular carcinoma screening among patients with hepatitis C and cirrhosis. Abbreviations: FFS, fee-for-service. Note: Follow-up period is defined as every 6-month period from the index date—the date of the first cirrhosis claim between 2014 and 2017.

The second sample comprised HCV patients with cirrhosis, regardless of DAA use. We defined an index date for each beneficiary as the date of the first cirrhosis claim between 2014 and 2017. We then followed up each patient for every 6-month period from the index date. Patients were required to be continuously enrolled in both Part A and Part B during each 6-month period. The presence of cirrhosis was identified from claims data by applying the CMS Chronic Conditions Data Warehouse algorithm [32], of ICD-9 code 571.5 or ICD-10 codes K74.0, K74.60, K74.69. Fig 2 presents a diagram of the second sample selection process.

### Quality measures

We measured quality of HCV care using measures from Medicare’s 2016 PQRS (S1 Table) and/or that were recommended by the American Association for the Study of Liver Diseases (AASLD) [7]: genotype or quantitative HCV ribonucleic acid (RNA) testing conducted within 12 months prior to the initiation of DAA treatment; quantitative HCV RNA testing within 6 months after the initiation of DAA treatment; and semi-annual screening for hepatocellular carcinoma (HCC) with ultrasound in adults with cirrhosis. For ease of interpretation, we considered tests conducted before DAA initiation (genotype or RNA testing) as “pre-treatment testing” and those performed after DAA initiation (RNA testing) as “post-treatment testing.”

RNA testing was identified by the following Current Procedural Terminology (CPT) codes: 87520, 87521, and 87522. Genotype testing was determined by CPT code 87902.

Ultrasound screening for HCC was defined by CPT codes 76700.

### Explanatory variables

Explanatory variables were the patient’s race/ethnicity, urban/rural residence, and socioeconomic status (SES). We categorized race/ethnicity into four groups: White, African-American, Hispanic, and other races (Asian/Pacific Islander, North American Native, and Unknown).

The rural-urban indicator was based on the patient’s ZIP code of residence using RUCA codes. To evaluate the effect of lacking access to specialists on disparities in the quality of HCV care in rural areas, we interacted rural-urban status with an indicator of underserved areas, defined as having less than one specialist per 2,000 population.

We measured patient SES by dual-eligibility status for Medicare and Medicaid (a joint federal and state program that provides health coverage to low-income individuals), ZIP-level median household income, and education. We divided income into tertiles and education into two groups: above and below the average percentage of the U.S. population who had attained at least a Bachelor’s degree.

### Covariates

We controlled for patient gender, age, and health risk (indicators for HIV/AIDs, cancer, cardiac diseases, diabetes, eye diseases, kidney diseases, drug and alcohol related disorder, and bone diseases). We included an indicator of cirrhosis in the first sample—i.e., DAA users—used to analyze pre- and post-treatment testing. We also controlled for region (Northeast, Midwest, South, and West) and total and rural populations aged 45 and over in each state because HCV infection is more prevalent in persons older than 40 years [33].

### Analytic methods

We used bivariate probit to estimate the probabilities of pre- and post-treatment testing, controlling for all covariates described above. Bivariate probit—a direct extension of univariate probit—is a joint model for two binary outcomes, assuming that the error terms in the two equations are correlated. This allows us to account for unobserved patient characteristics that affect both outcomes; for example, patients who sought pre-treatment testing might be more inclined to attend post-treatment testing. This will cause the errors in two univariate probit equations to be correlated, resulting in inefficient estimates. By estimating the correlation between the error terms, bivariate probit is not subject to this problem. Positive correlation implies that unmeasured factors leading patients to use pre-treatment testing also lead patients to use post-treatment testing. Negative correlation indicates an inverse relation between pre- and post-treatment testing.

We used multivariate logistic regression to estimate adjusted odds ratios (aOR) of undergoing semi-annual screening for HCC among patients with cirrhosis, controlling for all covariates described above, except cirrhosis, which is present in 100 percent of this sample. In addition, we included indicators for the patient’s state of residence and year to account for time-invariant state characteristics and annual differences.

We considered p < 0.05 as statistically significant in all analyses. Analyses were performed using SAS version 9.4 (SAS Institute, Cary, NC) and Stata version 15 (State-Corp LP, College Station, TX).

### Sensitivity analysis

Although the AASLD recommends that cirrhosis patients should be screened with ultrasound periodically, contrast-enhanced computed tomography (CT) and magnetic resonance imaging (MRI) are also standard diagnostic tests for HCC [34–37]. Hence, we used a separate logistic regression to estimate adjusted odds ratios of receiving semi-annual imaging tests, including ultrasound, contrast-enhanced CT, or MRI among cirrhosis patients.

## Results

### Pre- and post-treatment testing in DAA users

Table 1 presents characteristics of the study sample. A total of 41,800 beneficiaries met our inclusion criteria, of whom 93.47% and 84.99% received pre- and post-treatment testing, respectively. About 39% were female, 26% were African-American, 18% resided in rural areas, 66% were eligible for both Medicare and Medicaid, and the average age was 60.6 (this is consistent with previous studies showing that the majority of Medicare patients with HCV are under age 65 with disabililty) [38].

**Table 1:** Characteristics of Medicare beneficiaries who initiated direct-acting antiviral agents (DAA), 2014–2017.

	Overall	Pre-treatment testing^1^	No pre-treatment testing	*p-value*	Post-treatment testing^2^	No post-treatment testing	*p-value*
	(N = 41,800)	(N = 39,072)	(N = 2,728)		(N = 35,526)	(N = 6,274)	
	N/Mean	N/Mean	N/Mean		N/Mean	N/Mean	
	(%/SD)	(%/SD)	(%/SD)		(%/SD)	(%/SD)	
**Genotype/pre-treatment HCV RNA testing**	39,072 (93.47)						
**Post-treatment HCV RNA testing**	35,526 (84.99)						
**Female** *(ref*. *male)*	16,235 (38.84)	15,272 (39.09)	963 (35.30)	0.000	13,832 (38.93)	2,403 (38.30)	0.342
**Age**	59.73 (10.26)	59.76 (10.30)	59.39 (9.68)	0.670	60.03 (10.15)	58.08 (10.70)	0.000
**Race/ethnicity** *(ref*. *White)*				0.000			0.000
African American	10,813 (25.87)	10,020 (25.64)	793 (29.07)		9,055 (25.49)	1,728 (28.02)	
Hispanic	1,213 (2.90)	1,109 (2.84)	104 (3.81)		1,013 (2.85)	200 (3.19)	
Other	1,863 (4.46)	1,646 (4.21)	217 (7.95)		1,531 (4.31)	332 (5.29)	
**Socioeconomic characteristics**							
Per capita income *(tertile with ref*. *low)*				0.253			0.000
Medium	13,288 (31.79)	12,455 (31.88)	833 (30.54)		11,270 (31.72)	2,018 (32.16)	
High	13,924 (33.31)	12,982 (33.23)	942 (34.53)		12,156 (34.22)	1,768 (28.18)	
Education above average^3^	19,665 (47.05)	18,299 (46.83)	1,366 (50.07)	0.001	17,061 (48.02)	2,604 (41.50)	0.000
Dual eligibility status	27,667 (66.19)	25,826 (66.10)	1,841 (67.49)	0.139	23,058 (64.90)	4,609 (73.46)	0.000
**Geographic characteristics**							
Rural *(ref*. *urban)*	7,727 (18.49)	7,286 (18.65)	441 (16.17)	0.000	6,403 (18.02)	1,324 (21.10)	0.000
Low specialist density *(<1 specialist per 2*,*000 population)*	5,512 (13.19)	5,214 (13.34)	298 (10.92)	0.000	4,539 (12.78)	973 (15.51)	0.000
**Region** (*ref*. *Northeast*)				0.000			0.000
Midwest	7,806 (18.67)	7,214 (18.46)	592 (21.70)		6,561 (18.47)	1,245 (19.84)	
South	15,929 (38.11)	15,063 (38.55)	866 (31.74)		13,436 (37.82)	2,493 (39.74)	
West	8,921 (21.34)	8,283 (21.20)	638 (23.39)		7,724 (21.74)	1,197 (19.08)	
Total population aged 45 and older *(1*,*000*,*000 persons)*	5.16 (4.18)	5.15 (4.16)	5.34 (4.39)	0.019	5.20 (4.20)	4.99 (4.09)	0.000
Total rural population aged 45 and older *(1*,*000*,*000 persons)*	1.51 (1.62)	1.51 (1.61)	1.61 (1.78)	0.002	1.50 (1.60)	1.61 (1.72)	0.000
**Clinical Comorbidities**							
Cirrhosis	6,719 (16.07)	6,141 (15.72)	578 (21.19)	0.000	5,812 (16.36)	907 (14.46)	0.000
HIV/AIDS	2,136 (5.11)	1,882 (4.82)	254 (9.31)	0.000	1,834 (5.16)	302 (4.81)	0.247
Cancer	4,395 (10.51)	4,105 (10.51)	290 (10.63)	0.838	3,810 (10.72)	585 (9.32)	0.001
Diabetes	12,032 (28.78)	11,269 (28.84)	763 (27.97)	0.331	10.211 (28.74)	1,821 (29.02)	0.649
Cardiac disease	28,565 (68.34)	26,814 (68.63)	1,751 (64.19)	0.000	24,322 (68.46)	4,243 (67.63)	0.190
Eye disease	6,236 (14.92)	5,886 (15.06)	350 (12.83)	0.002	5,487 (15.45)	749 (11.94)	0.000
Bone disease	14,345 (34.32)	13,605 (34.82)	740 (27.13)	0.000	12,207 (20.05)	2,138 (34.08)	0.663
Kidney disease	8,599 (20.57)	7,956 (20.36)	643 (23.57)	0.000	7,122 (20.05)	1,477 (23.54)	0.000
Drug and alcohol related disorder	18,903 (45.22)	17,744 (45.41)	1,159 (42.49)	0.003	15,463 (43.53)	2,440 (54.83)	0.000

Abbreviations: HCV, Hepatitis C virus; RNA, Ribonucleic acid; HIV/AIDS, Human immunodeficiency virus/Acquired immunodeficiency syndrome

^1^ Pre-treatment testing includes receipt of genotype or quantitative HCV ribonucleic acid (RNA) testing within 12 months prior to direct-acting antiviral (DAA) initiation date

^2^ Pre-treatment testing includes receipt of quantitative HCV RNA testing within 6 months after DAA initiation date

^3^ Compared to average percentage of the U.S. population who attained at least a Bachelor’s degree

Table 2 presents the results from the bivariate probit regression. Patients in racial minority groups had a lower probability of receiving pre-treatment testing than whites. Being African-American, Hispanic, or other races lowered the probability of pre-treatment testing by 1.50 (95% CI: -2.10,-0.90), 1.60 (95% CI: -2.94,-0.26), and 4.54 percentage points (95% CI: -5.55,-3.54), respectively. Living in rural areas or counties with low specialist density was not associated with the probability of pre-treatment testing. The interaction term between rurality of residence and low specialist density suggests that specialist density did not significantly change the probability of pre-treatment testing in rural areas. Education was negatively associated with the probability of pre-treatment testing (Coef. = -0.06, 95% CI: -0.11, -0.00), whereas we observed no differences in the rates of pre-treatment testing among income tertiles.

**Table 2:** Estimated coefficients of bivariate probit model and marginal effects (N = 41,800).

	Pre-treatment testing^1^		Post-treatment testing^2^	
	Coef.	Marginal effect	Coef.	Marginal effect
	(95% CI)	(95% CI)	(95% CI)	(95% CI)
**Race/ethnicity**				
African American	-0.12***	-1.50 ***	-0.10***	-2.22***
	(-0.17,-0.07)	(-2.10, -0.90)	(-0.14,-0.06)	(-3.09, -1.35)
Hispanic	-0.13*	-1.60*	-0.09	-1.96
	(-0.24,-0.02)	(-2.94, -0.26)	(-0.17,0.00)	(-3.99, 0.06)
Other	-0.36***	-4.54***	-0.21***	-4.81***
	(-0.44,-0.28)	(-5.55, -3.54)	(-0.28,-0.14)	(-6.45, -3.17)
**Geographic characteristics**				
Rural	-0.03	-0.42	-0.09*	-2.09*
	(-0.13,-0.06)	(-1.60, 0.76)	(-0.16,-0.02)	(-3.76, -0.43)
Low specialist density *(1 specialist per 2*,*000 population)*	0.05	0.62	-0.04	-0.81
	(-0.01,0.11)	(-0.16,1.40)	(-0.08,0.01)	(-1.89,0.28)
Rural × Low specialist density	0.03	0.43	0.06	1.29
	(-0.09,0.16)	(-1.08,1.94)	(-0.04,0.15)	(-0.80,3.38)
**Socioeconomic characteristics**				
Per capita income (tertile)				
Medium	0.03	0.31	0.04	0.84
	(-0.03,0.08)	(-0.33,0.95)	(-0.00,0.08)	(-0.05,1.73)
High	0.03	0.37	0.07**	1.59**
	(-0.04,0.10)	(-0.47,1.20)	(0.02,0.12)	(0.39,2.80)
Education above average	-0.06*	-0.74*	0.04*	1.00*
	(-0.11,0.00)	(-1.43,-0.06)	(0.00,0.09)	(0.01,1.99)
Dual eligibility status	-0.01	-0.10	-0.12***	-2.71***
	(-0.26,-0.16)	(-0.65,0.45)	(-0.15,-0.08)	(-3.53,-1.90)
**Female**	0.03	1.42	0.01	0.28
	(-0.01,0.07)	(-0.08,0.92)	(-0.02,0.04)	(-0.44,1.00)
**Age**	0.00*	0.03*	0.00***	0.10***
	(0.00,0.00)	(0.00,0.06)	(0.00,0.01)	(0.06,0.14)
**Region**				
Midwest	-0.13***	-1.66***	-0.03	-0.78
	(-0.19,-0.07)	(-2.41,-0.91)	(-0.08,0.01)	(-1.89,0.33)
South	0.06*	0.72*	-0.03	-0.72
	(0.00,0.11)	(0.04,1.41)	(-0.07,0.01)	(-1.69,0.26)
West	-0.06**	-0.75**	0.00	0.04
	(-0.13,0.00)	(-1.56,0.06)	(-0.05,0.05)	(-1.17,1.24)
Total population aged 45 and older *(1*,*000*,*000 persons)*	-0.00	0.00	-0.00	0.00
	(-0.01,0.00)	(-0.01,0.00)	(-0.01,-0.00)	(-0.01,0.01)
Total rural population aged 45 and older *(1*,*000*,*000 persons)*	-0.02	-0.03	-0.03***	-0.07***
	(-0.03,-0.01)	(-0.04,-0.01)	(-0.04,-0.02)	(-0.09,-0.04)
**Clinical Comorbidities**				
Cirrhosis	-0.21***	-2.60***	0.08***	1.80***
	(-0.26,-0.16)	(-3.21,-1.99)	(0.04,0.12)	(0.84,2.76)
HIV/AIDS	-0.30***	-3.74***	0.11***	2.59***
	(-0.38,-0.23)	(-4.68,-2.80)	(0.04,0.18)	(0.98,4.19)
Cancer	-0.01	-0.09	0.05	1.16
	(-0.07,0.06)	(-0.88,0.70)	(0.00,0.10)	(-0.01,2.34)
Diabetes	0.04	0.56	0.01	-0.28
	(0.00,0.09)	(-0.01,1.13)	(-0.02,0.05)	(-0.54,1.09)
Cardiac disease	0.09***	1.13***	0.04*	0.88*
	(0.05,0.14)	(0.58,1.69)	(0.00,0.07)	(0.07,1.70)
Eye disease	0.07*	0.86*	0.10***	2.35***
	(0.01,0.12)	(0.15,1.56)	(0.06,0.15)	(1.32,3.37)
Bone disease	0.12***	1.45***	0.02	0.43
	(0.07,0.16)	(0.91,1.98)	(-0.01,0.05)	(-0.31,1.18)
Kidney disease	-0.11***	-1.34***	-0.12***	-2.64***
	(-0.16,-0.06)	(-1.95,-0.72)	(-0.15,-0.08)	((-3.53,-1.74)
Drug and alcohol related disorder	0.05*	0.65*	-0.19***	-4.41***
	(0.01,0.09)	(0.13,1.16)	(-0.23,-0.16)	(-5.15,-3.67)
rho	0.34***			
	(0.31,0.36)			

Abbreviations: HIV/AIDS, Human immunodeficiency virus/Acquired immunodeficiency syndrome

Note: Marginal effects were reported as percentage points

^1^ Pre-treatment testing includes receipt of genotype or quantitative HCV ribonucleic acid (RNA) testing within 12 months prior to direct-acting antiviral (DAA) initiation date

^2^ Pre-treatment testing includes receipt of quantitative HCV RNA testing within 6 months after DAA initiation date

*p<0.05,

**p<0.01,

***p<0.001

The key independent variables had similar effects on the probability of post-treatment testing. The association between racial/ethnic minorities and post-treatment testing remained negative and significant. In contrast to pre-treatment testing, patients residing in rural areas were less likely to have post-treatment testing than those living in urban areas (Coef. = -0.06, 95% CI: -0.11, -0.01). The marginal effect indicates that the probability of post-treatment testing decreased by 2.09 percentage points (95% CI: -3.76, -0.43) for patients living in rural areas. In addition, the results show that both income and education were associated with a higher probability of post-treatment testing (Coef. = 0.07, 95% CI: 0.02, 0.12 and Coef. = 0.04, 95% CI: 0.00, 0.09, respectively). The marginal effects were 1.59 and 1.00 percentage points, respectively. Dual eligibility status significantly reduced the probability of post-treatment testing by 2.71 percentage points (95% CI: -3.53, -1.90).

The probabilities of pre- and post-treatment testing increased by 0.03 (95% CI: 0.00, 0.06) and 0.10 percentage points (95% CI: 0.06, 0.14) for each 1-year increase in age, respectively. The presence of some comorbidities (e.g. cardiac or eye diseases) was associated with increased probabilities of pre- and post-treatment testing, while having kidney diseases was negatively associated with both tests.

The correlation coefficient shown in Table 2 indicates whether unobserved factors that influence the probability of testing before DAA treatment also affect the probability of testing after treatment. The significant and positive correlation (rho = 0.34, *p*<0.000) implies that the probabilities of pre- and post-treatment testing were positively correlated.

### Routine screening for HCC in patients with cirrhosis

A total of 60,350 beneficiaries (200,319 observations) met our study inclusion criteria: 70.3% had at least two follow-up periods and 39.8% received at least one semi-annual HCC screening during the study period (S2 Table). Among individuals with at least two follow-up periods, the proportion of those receiving at least two; only one; and no ultrasound are reported in S2 Table.

Table 3 exhibits descriptive data of the study variables for the analysis of HCC screening among HCV patients with cirrhosis. Of the 200,319 observations, 19.4% underwent semi-annual screening for HCC with ultrasound. The following presents descriptive statistics of beneficiary characteristics across all follow-up periods: about 39% were female, 15% were African-American, 15% resided in rural areas, 55% were eligible for both Medicare and Medicaid, and the average age was 63.65. Receipt of semi-annual HCC screening was positively associated with being female, living in urban areas or neighborhoods with higher income and education, and having comorbid conditions, such as cancer, diabetes, cardiac disease, eye diseases, bone diseases, and kidney diseases.

**Table 3:** Descriptive data for the analysis of semi-annual hepatocellular carcinoma (HCC) screening with ultrasound among Medicare beneficiaries with hepatitis C and cirrhosis.

	Overall	HCC screening	No HCC screening	*p-value*
	(N = 200,319)	(N = 38,879)	(N = 161,440)	
	N/Mean	N/Mean	N/Mean	
	(%/SD)	(%/SD)	(%/SD)	
**Female** *(ref*. *male)*	71,871 (35.88)	15,182 (39.05)	56,689 (35.11)	0.000
**Age**	63.51 (9.13)	63.65 (9.07)	63.48 (9.14)	0.000
**Race/ethnicity** *(ref*. *White)*				
African American	33,181 (16.56)	5,825 (14.98)	27,356 (16.95)	0.001
Hispanic	9,629 (4.81)	2,362 (6.08)	7,267 (4.50)	0.000
Other	12,338 (6.16)	2,686 (6.91)	9,652 (5.98)	0.000
**Socioeconomic characteristics**				
Per capita income (tertile)				
Medium	63,239 (31.57)	11,672 (30.02)	51,567 (31.94)	0.000
High	66,162 (33.03)	13,580 (34.93)	52,582 (32.57)	0.000
Education above average	95,302 (47.58)	19,113 (49.16)	76,189 (47.19)	0.000
Dual eligibility status	106,554 (53.19)	21,302 (54.79)	85,252 (52.81)	0.000
**Geographic characteristics**				
Rural *(ref*. *urban)*	33,489 (16.72)	5,640 (14.51)	27,849 (17.25)	0.000
Low specialist density *(1 specialist per 2*,*000 population)*	52,872 (26.39)	9,176 (23.60)	43,696 (27.07)	0.000
**Region** (*ref*. *Northeast*)				
Midwest	33,667 (16.81)	4,772 (12.27)	28,895 (17.90)	0.000
South	82,316 (41.09)	14,601 (37.55)	67,715 (41.94)	0.000
West	47,834 (23.88)	11,873 (30.54)	35,961 (22.28)	0.000
Total population aged 45 and older *(1*,*000*,*000 persons)*	5.77 (4.41)	6.38 (4.84)	5.62 (4.29)	0.000
Total rural population aged 45 and older *(1*,*000*,*000 persons)*	1.54 (1.55)	1.51 (1.64)	1.54 (1.52)	0.002
**Clinical Comorbidities**				
HIV/AIDS	5,428 (2.71)	1,033 (2.66)	4,395 (2.72)	0.476
Cancer	27,521 (13.74)	5,569 (14.32)	21,952 (13.60)	0.000
Diabetes	85,995 (42.93)	17,369 (44.67)	68,626 (42.51)	0.000
Cardiac disease	153,788 (76.77)	30,612 (78.74)	123,176 (76.30)	0.000
Eye disease	30,252 (15.10)	6,848 (17.61)	23,404 (14.50)	0.000
Bone disease	72,942 (36.41)	14,590 (37.53)	58,352 (36.14)	0.000
Kidney disease	87,007 (43.43)	17,977 (46.24)	69,030 (42.76)	0.000
Drug and alcohol related disorder	109,648 (54.74)	20,852 (53.63)	88,796 (55.00)	0.000

Abbreviations: HCC, Hepatocellular carcinoma; HCV, Hepatitis C virus; RNA, Ribonucleic acid; HIV/AIDS, Human immunodeficiency virus/Acquired immunodeficiency syndrome

Table 4 shows the results from the multivariate logistic regression. Being African-American significantly lowered with the odds of HCC screening by 7% (aOR = 0.93, 95% CI: 0.90, 0.96), whereas the odds of screening for Hispanics were higher than the odds for whites (aOR = 1.09, 95% CI: 1.04, 1.15).

**Table 4:** Adjusted odds ratios of undergoing semi-annual hepatocellular carcinoma screening with ultrasound among Medicare beneficiaries with hepatitis C and cirrhosis (N = 200,319).

	Odds Ratio	95% CI	*p*
**Race/ethnicity** *(ref*. *White)*			
African American	0.93	(0.90–0.96)	0.000
Hispanic	1.09	(1.04–1.15)	0.001
Other	0.99	(0.94–1.04)	0.646
**Geographic characteristics**			
Rural *(ref*. *urban)*	0.86	(0.80–0.92)	0.000
Low specialist density *(1 specialist per 2*,*000 population)*	0.93	(0.90–0.97)	0.000
Rural × Low specialist density	1.09	(1.01–1.19)	0.032
**Socioeconomic characteristics**			
Per capita income (tertile)			
Medium	0.98	(0.95–1.01)	0.141
High	1.02	(0.98–1.06)	0.401
Education above average	1.01	(0.98–1.05)	0.420
Dual eligibility status	1.01	(0.99–1.04)	0.415
**Female** *(ref*. *male)*	1.17	(1.14–1.20)	0.000
**Age**	1.00	(0.99–1.00)	0.557
**Region** (*ref*. *Northeast*)			0.000
Midwest	0.58	(0.10–3.53)	
South	1.02	(0.29–3.55)	0.972
West	0.75	(0.50–1.13)	0.175
Total population aged 45 and older *(1*,*000*,*000 persons)*	1.06	(0.94–1.19)	0.330
Total rural population aged 45 and older *(1*,*000*,*000 persons)*	1.11	(0.22–5.70)	0.899
**Clinical Comorbidities**			
HIV/AIDS	0.96	(0.90–1.03)	0.293
Cancer	1.06	(1.03–1.10)	0.001
Diabetes	0.99	(0.97–1.02)	0.597
Cardiac disease	1.12	(1.09–1.16)	0.000
Eye disease	1.20	(1.16–1.24)	0.000
Bone disease	1.03	(1.01–1.06)	0.009
Kidney disease	1.15	(1.13–1.18)	0.000
Drug and alcohol related disorder	0.96	(0.94–0.98)	0.001

Abbreviations: HIV/AIDS, Human immunodeficiency virus/Acquired immunodeficiency syndrome

The odds of semi-annual HCC screening were 14% lower in patients from rural areas (aOR = 0.86, 95% CI: 0.80, 0.92) than those residing in urban areas. Living in areas with low specialist density reduced the odds of HCC screening by 7% (aOR = 0.93, 95% CI: 0.90, 0.97). Low specialty physician density was associated with higher odds of receiving routine screening for HCC in rural areas, compared with urban areas (aOR = 1.09, 95% CI: 1.01, 1.19).

ZIP-level income, education, or dual eligibility did not significantly change the odds of receiving semi-annual HCC screening with ultrasound.

Several patient factors were significantly associated with HCC screening. The presence of certain comorbidities, such as cancer, cardiac diseases, eye diseases, and bone diseases, and kidney disease was associated with increased odds of semi-annual HCC screening, whereas those with drug and alcohol related disorder had lower odds.

### Sensitivity analysis

The findings from the sensitivity analysis of semi-annual HCC screening with diagnostic imaging (including ultrasound, MRI, and CT scan) were similar to the main analysis (S3 Table). Living in rural areas reduced the odds by 14% (aOR = 0.86, 95% CI: 0.81, 0.91), while Hispanics were associated with higher odds. Although African-Americans had lower odds of ultrasound screening, we did not find a Black-white gap in diagnostic imaging for HCC (aOR = 0.98, 95% CI: 0.95, 1.00). In contrast to the main analysis, ZIP-level income and education were positively associated with HCC screening with diagnostic imaging, while dual eligibility lowered the odds. Compared with those in the lowest income tertile, individuals in areas with the highest income tertile had 8% higher odds (aOR = 1.08, 95% CI: 1.04, 1.12). Those living in areas with a higher percentage of college graduates also had increased odds of HCC screening with diagnostic imaging (aOR = 1.06, 95% CI: 1.03, 1.09). Individuals who were eligible for both Medicare and Medicaid had 9% lower odds (aOR = 0.91, 95% CI: 0.90, 0.93).

## Discussion

Quality of HCV care differed by patient race/ethnicity. This result is consistent with previous studies, which showed that patients of minority race/ethnicity were less likely to receive recommended HCV care and treatment [10, 12, 22]. Patients in racial minority groups had a lower probability of receiving pre- and post-treatment testing than whites. In addition, the odds of undergoing HCC screening every 6 months were lower among African-American patients, while Hispanics had higher odds, compared to whites. These findings suggest that more effort is needed to achieve equitable quality of HCV care across all race/ethnicity groups.

Although rurality of residence was not associated with lower use of pre-treatment testing, we found that rural patients were less likely to receive testing after DAA treatment than their urban counterparts. Rural communities have long struggled to improve the quality of health care [39, 40]. Shortages of physicians and other health care professionals have been identified a major factor contributing to poor quality care in rural areas [39]. Thus, we expected that obtaining high-quality health care services would be more challenging for patients living in rural areas with relatively low specialist density. However, the results suggest that specialist density did not significantly change the probabilities of receiving recommended pre- and post-treatment testing in rural areas.

Similarly, we found that rural HCV patients diagnosed with cirrhosis had lower odds of semi-annual HCC screening than those in urban areas. Since HCC screening for adults with cirrhosis requires a referral to a specialist, low specialist density was associated with lower odds of semi-annual HCC screening. However, low specialty physician density increased the odds of routine screening for HCC in rural areas, compared with urban areas. This interaction suggests that quality of HCV care in rural areas could be explained by factors other than a shortage of specialists.

A variety of elements contribute to lower quality of care in rural areas, including shortages of medical equipment and services, poor health literacy, lack of transportation, and travel time and long distances [13, 39]. Rural hospitals or local outpatient clinics might not have sufficient equipment and services to perform needed genotype and viral load tests. Health literacy—the ability to understand health information—is a substantial concern in rural communities due to low educational attainment [13]. With limited health literacy, patients may find it challenging to communicate with healthcare professionals, follow their instructions, and navigate the healthcare system [41, 42]. Finally, traveling to receive medical services might place a heavy burden on rural patients, especially those with low incomes, no paid time off work, physical limitations, or no personal transportation [13].

Patient SES and neighborhood characteristics were associated with disparities in quality of HCV care. The negative and significant association between dual eligibility status and post-treatment testing suggests that low-income patients may find it challenging to receive the recommended HCV tests after initiation of DAA. In addition, the results for income and education imply that socio-economically disadvantaged patients have limited adherence to recommended HCV care. Beneficiaries living in areas with higher income and a higher percentage of college graduates were more likely to receive post-treatment testing and semi-annual HCC screening. These results indicate that differences in health literacy and affordability of health care might contribute to the disparities in quality of HCV care associated with patient SES.

### Limitations

Several limitations need to be recognized in interpreting our findings. First, we selected patients who used HCV care after a one-year washout period; hence, the proportion of patients having genotype or pre-treatment testing may be underestimated if some patients received HCV care prior to the washout period and had those tests performed during their earlier HCV care. Second, due to the limitations of Medicare claims data, we could not assess other recommended HCV care indicators (such as review of current medications and treatment options, liver fibrosis stage, etc.). Third, our study had a limited follow-up period for HCC screening. Future studies are needed to examine factors associated with the utilization of HCC surveillance using data from recent years. Finally, our study was limited to FFS Medicare beneficiaries; thus, the results may not generalize to other populations, such as Medicare Advantage enrollees, Medicaid beneficiaries (except those dually eligible for Medicare), and patients with commercial insurance.

### Conclusions and implications

Disparities in quality of HCV care among Medicare beneficiaries existed by patient race/ethnicity, urban/rural residence, and socioeconomic status. Continued efforts are needed to improve quality of care for all HCV patients—especially rural patients and racial/ethnic minorities.

## Supporting information

S1 Table 2016 Physician quality reporting system measures. (DOCX)

S2 Table Proportion of Medicare beneficiaries with hepatitis C and cirrhosis underwent semi-annual hepatocellular carcinoma surveillance. (DOCX)

S3 Table Adjusted odds ratios of undergoing semi-annual hepatocellular carcinoma screening with diagnostic imaging among Medicare beneficiaries with hepatitis C and cirrhosis (N = 200,319). (DOCX)
