# Supplementary material for: Disparities in the quality of care for chronic hepatitis C among Medicare beneficiaries
Source: PLoS One. 2022 Mar 10;17(3):e0263913. doi: 10.1371/journal.pone.0263913 (PMC8912154; doi:10.1371/journal.pone.0263913)
Supplement: S1 Table — (DOCX) [file pone.0263913.s001.docx]

**S1 Table. 2016 Physician Quality Reporting System Measures**

| **Measure Title** | **Measure Description** |
| --- | --- |
| Hepatitis C: Hepatitis C Virus (HCV) Genotype Testing Prior to Treatment^1^ | Percentage of patients aged 18 years and older with a diagnosis of chronic hepatitis C who started antiviral treatment within the 12 month reporting period for whom hepatitis C virus (HCV) genotype testing was performed within 12 months prior to initiation of antiviral treatment |
| Hepatitis C: Ribonucleic Acid (RNA) Testing Before Initiating Treatment^2^ | Percentage of patients aged 18 years and older with a diagnosis of chronic hepatitis C who started antiviral treatment within the 12 month reporting period for whom quantitative hepatitis C virus (HCV) ribonucleic acid (RNA) testing was performed within 12 months prior to initiation of antiviral treatment |
| Hepatitis C: Hepatitis C Virus (HCV) Ribonucleic Acid (RNA) Testing Between 4-12 Weeks After Initiation of Treatment^3^ | Percentage of patients aged 18 years and older with a diagnosis of chronic hepatitis C who are receiving antiviral treatment for whom quantitative hepatitis C virus (HCV) ribonucleic acid (RNA) testing was performed between 4-12 weeks after the initiation of antiviral treatment |

^1^ Hepatitis C: Hepatitis C Virus (HCV) Genotype Testing Prior to Treatment. (2021). CMS Measures Inventory Tool. Retrieved January 6, 2022, from <https://cmit.cms.gov/CMIT_public/ViewMeasure?MeasureId=394>

^2^ Hepatitis C: Ribonucleic Acid (RNA) Testing Before Initiating Treatment. (2021). CMS Measures Inventory Tool. Retrieved January 6, 2022, from <https://cmit.cms.gov/CMIT_public/ReportMeasure?measureRevisionId=109>

^3^ Hepatitis C: Hepatitis C Virus (HCV) Ribonucleic Acid (RNA) Testing Between 4-12 Weeks After Initiation of Treatment. (2021). CMS Measures Inventory Tool. Retrieved January 6, 2022, from <https://cmit.cms.gov/CMIT_public/ReportMeasure?measureRevisionId=111>
