# Supplementary material for: Disparities in the quality of care for chronic hepatitis C among Medicare beneficiaries
Source: PLoS One. 2022 Mar 10;17(3):e0263913. doi: 10.1371/journal.pone.0263913 (PMC8912154; doi:10.1371/journal.pone.0263913)
Supplement: S2 Table — (DOCX) [file pone.0263913.s002.docx]

**S2 Table. Proportion of Medicare Beneficiaries with Hepatitis C and Cirrhosis Underwent Semi-annual Hepatocellular Carcinoma Surveillance**

| Number of Medicare beneficiaries with hepatitis C and cirrhosis having at least 1 follow-up periods | 60,350 (100%) |
| --- | --- |
| Received at least 2 ultrasound | 8,661 (14.35% |
| Received only 1 ultrasound | 15,360 (25.45%) |
| Received no ultrasound | 36,329 (60.20%) |
| Number of Medicare beneficiaries with hepatitis C and cirrhosis having at least 2 follow-up periods | 42,402 (100%) |
| Received at least 2 ultrasound | 8,661 (20.43%) |
| Received only 1 ultrasound | 11,003 (25.95%) |
| Received no ultrasound | 22,738 (53.62%) |

Note: Follow-up period is defined as every 6-month period from the index date – the date of the first cirrhosis claim between 2014 and 2017.
