# Supplementary material for: Disparities in the quality of care for chronic hepatitis C among Medicare beneficiaries
Source: PLoS One. 2022 Mar 10;17(3):e0263913. doi: 10.1371/journal.pone.0263913 (PMC8912154; doi:10.1371/journal.pone.0263913)
Supplement: S3 Table — (DOCX) [file pone.0263913.s003.docx]

**S3 Table. Adjusted Odds Ratios of Undergoing Semi-annual Hepatocellular Carcinoma Screening with Diagnostic Imaging among Medicare Beneficiaries with Hepatitis C and Cirrhosis (N=200,319)**

|  | Odds Ratio | 95% CI | *p* |
| --- | --- | --- | --- |
| **Race/ethnicity** *(ref. White)* |  |  |  |
| African American | 0.98 | (0.95-1.00) | 0.087 |
| Hispanic | 1.10 | (1.05-1.15) | 0.000 |
| Other | 1.19 | (1.15-1.24) | 0.000 |
| **Geographic characteristics** |  |  |  |
| Rural *(ref. urban)* | 0.86 | (0.81-0.91) | 0.000 |
| Low specialist density  *(1 specialist per 2,000 population)* | 0.95 | (0.92-0.98) | 0.001 |
| Rural $\times$Low specialist density | 1.07 | (1.00-1.15) | 0.056 |
| **Socioeconomic characteristics** |  |  |  |
| Per capita income (tertile) |  |  |  |
| Medium | 1.00 | (0.98-1.03) | 0.794 |
| High | 1.08 | (1.04-1.12) | 0.000 |
| Education above average | 1.06 | (1.03-1.09) | 0.000 |
| Dual eligibility status | 0.91 | (0.90-0.93) | 0.000 |
| **Female** *(ref. male)* | 1.05 | (1.02-1.07) | 0.000 |
| **Age** | 1.00 | (1.00-1.00) | 0.001 |
| **Region** (*ref. Northeast*) |  |  |  |
| Midwest | 0.37 | (0.08-1.74) | 0.209 |
| South | 0.45 | (0.15-1.30) | 0.141 |
| West | 0.54 | (0.38-0.76) | 0.000 |
| Total population aged 45 and older  *(1,000,000 persons)* | 1.10 | (1.00-1.22) | 0.062 |
| Total rural population aged 45 and older  *(1,000,000 persons)* | 1.53 | (0.38-6.23) | 0.553 |
| **Clinical Comorbidities** |  |  |  |
| HIV/AIDS | 0.86 | (0.81-0.92) | 0.000 |
| Cancer | 1.08 | (1.05-1.11) | 0.000 |
| Diabetes | 0.99 | (0.97-1.01) | 0.210 |
| Cardiac disease | 1.10 | (1.08-1.13) | 0.000 |
| Eye disease | 1.21 | (1.18-1.24) | 0.000 |
| Bone disease | 0.98 | (0.96-1.00) | 0.074 |
| Kidney disease | 1.04 | (1.02-1.06) | 0.000 |
| Drug and alcohol related disorder | 0.91 | (0.89-0.93) | 0.000 |

Abbreviations: HIV/AIDS, Human immunodeficiency virus/Acquired immunodeficiency syndrome
